# So Far Away, Yet So Close: Strong Genetic Structure in *Homonota uruguayensis* (Squamata, Phyllodactylidae), a Species with Restricted Geographic Distribution in the Brazilian and Uruguayan Pampas

**DOI:** 10.1371/journal.pone.0118162

**Published:** 2015-02-18

**Authors:** Jéssica F. Felappi, Renata C. Vieira, Nelson J. R. Fagundes, Laura V. Verrastro

**Affiliations:** 1 Departamento de Zoologia, Universidade Federal do Rio Grande do Sul, Porto Alegre, Brazil; 2 Departamento de Genética, Universidade Federal do Rio Grande do Sul, Porto Alegre, Brazil; University of Exeter, UNITED KINGDOM

## Abstract

The Pampas is a biologically rich South American biome, but is poorly represented in phylogeographic studies. While the Pleistocene glacial cycles may have affected the evolutionary history of species distributed in forested biomes, little is known about their effects on the habitats that remained stable through glacial cycles. The South American Pampas have been covered by grasslands during both glacial and interglacial periods and therefore represent an interesting system to test whether the genetic structure in such environments is less pronounced. In this study, we sampled Pampean populations of *Homonota uruguayensis* from Southern Brazil and Uruguay to assess the tempo and mode of population divergence, using both morphological measurements and molecular markers. Our results indicate that, in spite of its narrow geographic distribution, populations of *H. uruguayensis* show high levels of genetic structure. We found four major well-supported mtDNA clades with strong geographic associations. Estimates of their divergence times fell between 3.16 and 1.82 million years before the present. Populations from the central portion of the species distribution, on the border between Uruguay and Brazil, have high genetic diversity and may have undergone a population expansion approximately 250,000 years before the present. The high degree of genetic structure is reflected in the analyses of morphological characters, and most individuals could be correctly assigned to their parental population based on morphology alone. Finally, we discuss the biogeographic and conservation implications of these findings.

## Introduction

In spite of its rich biological diversity, South America is one of the least studied continents in terms of its phylogeography [1]. Phylogeography is helpful for revealing the patterns of evolutionary lineages among taxa and allows researchers to infer the processes behind such patterns [2]. Considering the different biomes or ecoregions within South America, it is no surprise that most studies are in the Amazon, the Andes, and the Brazilian Atlantic forest. The high biological diversity in these biomes is seen as an opportunity to test refuge hypotheses associated with climatic oscillations in the Quaternary [3]. In general, even though the Quaternary glacial cycles might have impacted the spatial distribution of genetic lineages by restricting some species or populations into glacial refugia (e.g., [4], [5]), these studies have shown that the Neogene orogenic events were also fundamental for generating the biological (including genetic) diversity of the South American biomes [3]. On the other hand, little is known about the potential impact of Pleistocene climatic oscillations on species occurring in environments dominated by grasslands in both glacial and interglacial periods, such as the Pampas [6].

The “Pampas”, also known as the Rio de la Plata grasslands [7], is one of the largest temperate grasslands in the world, covering approximately 760,000 km^2^ in Central-Eastern Argentina, Uruguay, and Southern Brazil [8]. Most of its vegetation consists of herbaceous plants, but the region is far from homogenous. The Pampas has a complex geological history that has resulted in a wide range of soil types, which in turn result in different plant communities [9], [10]. Because of such heterogeneity, several authors have proposed dividing this area into smaller “provinces” or “regions” (see e.g., [9], [11]). Nonetheless, in this study, we use the term “Pampas” inclusively [12]. Importantly, the Pampas is underrepresented in conservation policies. In Brazil, it has lost approximately 25% of its area due to the expansion of agriculture and silviculture, overgrazing, and the introduction of invasive species for pasture [11]. Despite these current threats and its biological richness, few studies have examined the phylogeographic patterns in the Pampas. Strong genetic structure was found in *Ctenomys riograndensis*, a fossorial mammal, which may have arisen after a recent population expansion [13]. On the other hand, studies of *Turnera sidoides* and *Petunia axillaris*, two herbaceous plant species, revealed a weak genetic structure, possibly associated with events in the last 100,000 years [14], [15], as did a recent study on *Conepatus chinga*, a terrestrial mammal [16].

**Fig 1:**
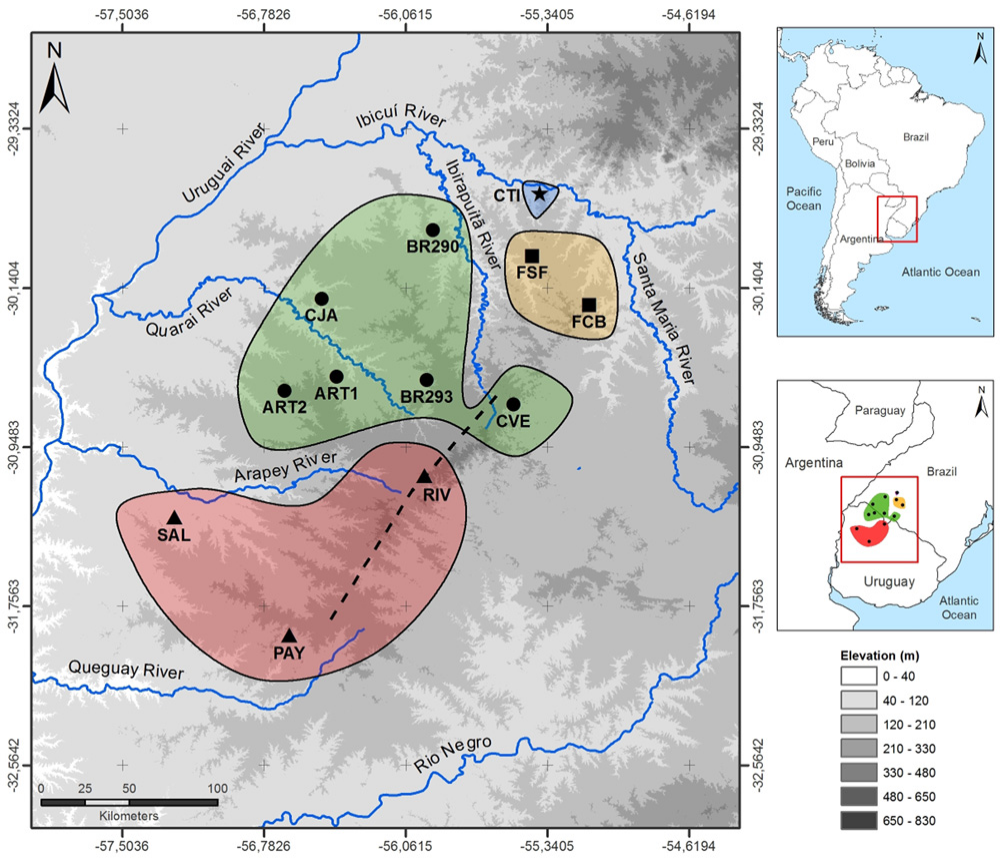
Sample sites for *Homonota uruguayensis* individuals used in this study. **Codes for the sampled populations are shown according to** Table 1. Major mtDNA clades (see text for details) are represented by stars (Clade I), triangles (Clade II), squares (Clade III), and circles (Clade IV) and delimited geographically by a solid line coloured according to each major clade (Clade I—blue; Clade II—red; Clade III—orange; Clade IV—green). The dotted line represents the Haedo Range, in Uruguay, and “Coxilha Grande”, in Brazil. Elevation is shown in grayscale according to the legend. The Uruguay River form parts of the boundaries of Argentina, Brazil, and Uruguay, while the Quaraí River forms part of the boundaries of Brazil and Uruguay.

*Homonota uruguayensis* (Vaz-Ferreira & Sierra de Soriano, 1961) is a small lizard (smaller than 50 mm snout-to-vent) belonging to the Phyllodactylidae family of Neotropical geckos [17]. This species occurs in the Pampas in northwestern Uruguay and southwestern Brazil and is associated with rocky outcrops used for shelter and nesting [18] (Fig. 1). A recent molecular phylogenetic study suggested that *H*. *uruguayensis* is part of the “borellii group”, a sister clade to that of *H*. *borellii*, *H*. *rupicola*, and *H*. *taragui* [19]. The same study also suggested that marine transgressions that occurred in the Late Miocene (5 to 10 million years ago) might have played a role in the history of this group by isolating an ancestral population in the Uruguayan Pampas that later expanded towards Argentina [19]. *H*. *uruguayensis* has been subject to a number of ecological studies [20]-[25] that have shown that suitable areas for this species depends not only on the presence of rocky outcrops, but also on the size of the rocks that are available as nests. The effect of such demanding habitat requirements on the genetic structure among populations is unknown.

The Pampas have been dominated by grasslands during both glacial and interglacial periods [6], and therefore one can ask whether such stability would be reflected in a lower genetic structure among populations, as predicted by the climate refuge hypothesis (e.g., [4], [5]). Alternatively, Pampean rivers may have acted as drivers of population divergence, as predicted by the “rivers as barriers” hypothesis (e.g., [26]). For example, in the first scenario, we also expect that *H*. *uruguayensis* populations exhibit stable population size throughout the Pleistocene and a relatively homogeneous genetic structure shaped by isolation by distance. In the second scenario, however, phylogeographic structure among populations may be greater, with the main intraspecific clades being associated with major rivers in the landscape. *H*. *uruguayensis* is an interesting model system to test these hypotheses as the strong ecological requirements of this species may limit dispersal among populations and lead to population differences in the absence of physical barriers or climatic oscillations. In this study, we take a phylogeographic approach to better understand the evolutionary history of *H*. *uruguayensis*, using molecular markers and morphological measures. More specifically, we aim to answer the following questions: How strong is the genetic structure in this species and what is the temporal depth of the mtDNA genealogy? Is there evidence of changes to this species’ population size during the Pleistocene? Is morphological variation within this species concordant with the levels of population structure suggested by genetic data?

## Material and Methods

### Sample Collection

We collected 106 individuals of *H*. *uruguayensis* from 12 localities (Table 1; Fig. 1) in Brazil and Uruguay covering the entire known distribution for this species. Geographic distances among sample sites range from 25 km to 275 km. Dr. Mariana Morando (CENPAT—Puerto Madryn, Argentina) kindly provided DNA samples from one individual of *H*. *fasciata* Duméril & Bibron 1836 and *H*. *borellii* Peracca 1897, collected from the Argentinean provinces of Santiago del Estero and Mendoza, respectively. These two species were used as outgroups.

**Table 1:** Sample localities and genetic diversity for the surveyed populations of ***H*. *uruguayensis***.

Population (Country)	Lat. (S)	Long. (W)	N	S	h	Hd +/- S.D.	π +/- S.D.	D	Fs
FCB—Fazenda Casa Branca (BR)	-30.2275	-55.1269	10	4	2	0.20 +/- 0.15	0.0007 +/- 0.0006	-1.67*	1.74
FSF—Fazenda São Francisco (BR)	-29.9783	-55.4164	2	3	2	1.00 +/- 0.50	0.0027 +/- 0.0031	0.00	1.10
CTI—Cerro do Tigre (BR)	-29.6603	-55.3781	10	7	5	0.71 +/- 0.12	0.0015 +/- 0.0011	-1.38	0.19
BR290—Alegrete BR290 (BR)	-29.8458	-55.9208	10	4	3	0.51 +/- 0.16	0.0009 +/- 0.0007	-1.56*	0.39
BR293—Livramento BR293 (BR)	-30.6081	-55.9536	11	16	6	0.87 +/- 0.07	0.0065 +/- 0.0037	1.23	1.63
CVE—Cerros Verdes (BR)	-30.7322	-55.5118	10	19	10	1.00 +/- 0.04	0.0051 +/- 0.0030	-0.70	-5.49*
CJA—Cerro do Jarau (BR)	-30.1947	-56.4883	10	7	4	0.78 +/- 0.09	0.0033 +/- 0.0021	1.89	2.01
ART1—Artigas 1 (UY)	-30.5919	-56.4119	9	14	7	0.92 +/- 0.09	0.0057 +/- 0.0034	1.11	-0.75
ART2—Artigas 2 (UY)	-30.6625	-56.6764	8	7	2	0.25 +/- 0.18	0.0016 +/- 0.0012	-1.64*	3.20
RIV—Rivera (UY)	-31.0943	-55.9650	6	11	3	0.60 +/- 0.21	0.0039 +/- 0.0026	-0.89	2.58
PAY—Paysandu (UY)	-31.9043	-56.6539	10	3	5	0.64 +/- 0.15	0.0010 +/- 0.0008	0.10	-0.70
SAL—Salto (UY)	-31.3046	-57.2384	10	0	1	0	0.00000	-	-
All populations	-	-	106	149	50	0.97 +/- 0.01	0.0262 +/- 0.0128	0.02	-1.01

BR—Brazil; UY—Uruguay; Lat.—Latitude; Long.—Longitude; N—Number of individuals; S—number of segregating sites; h—number of haplotypes; Hd—haplotype diversity; S.D.—standard deviation; π—nucleotide diversity; D—Tajima’s D; Fs—Fu’s Fs.

*P<0.05 or*P<0.02 for Tajima’s D or Fu’s FS, respectively.

### Ethics Statement

Collection of *H*. *uruguayensis* specimens was approved by the governmental authorities of both countries (Ministério do Meio Ambiente, Brazil—SISBIO 12613–1 and Dirección de Recursos Naturales del Ministerio de Ganadería, Agricultura y Pesca, Uruguay). Euthanasia of all specimens was performed by injecting 2mg of 10% ketamine hydrochloride close to the animal’s heart (http://www.asih.org/sites/default/files/documents/resources/guidelinesherpsresearch2004.pdf). The abovementioned permits include the method of euthanasia employed in this study. Specimens collected in Brazil were deposited in the Herpetology Laboratory’s scientific collection at the Federal University of Rio Grande do Sul. Specimens collected in Uruguay were deposited in the scientific reptile collection of the Faculty of Sciences, at the National Museum of Natural History in Montevideo. Currently, *H*. *uruguayensis* is not designated as an endangered species in any list.

### Molecular Methods

Tissue from the liver, tail muscle or tongue was used for DNA extraction. Immediately after collection, individuals were preserved in absolute ethanol and then stored at-20°C. DNA was extracted using a salting-out method as in Medrano *et al*. [27]. We studied two fragments of the mitochondrial DNA (720bp of the cytochrome *b* gene—cyt*b*, and 420bp of the 12S rDNA gene—12S), which were amplified by Polymerase Chain Reaction (PCR) using the primers 703Botp (5’TCA AAY ATC TCA ACC TGA TGA AAY TTY GG3’) and MVZ16p (5’GGC AAA TAG GAA GTA TCA YTC TGG YTT3’) as modified from Pook, Wüster & Thorpe [28], and L1091 (5’AAA CTR GGA TTA GAT ACC CYA CTAT3’) and H1478 (5’GAG GGT GAC GGG CGG TGT GT3’) as modified from Kocher *et al*. [29] for cyt*b* and 12S, respectively. For all reactions, we used 1.5 mM MgCl2, 1X PCR Buffer, 0.2 μM of each dNTP, 0.2 μM of each primer, and 0.2 U Taq DNA polymerase. We used the cycling conditions suggested by Carranza *et al*. [30] with an annealing temperature of 56°C. PCR products were checked in 1% agarose gel stained with GelRed^TM^, purified with exonuclease I and shrimp alkaline phosphatase (ExoSAP, GE Healthcare) and Sanger sequenced in both directions at Macrogen (Seoul, South Korea).

### Sequence Analysis

Chromatograms were visually inspected in the software Chromas v.2.33 (http://www.technelysium.com.au) and manually edited using MEGA 5 [31]. The cyt*b* gene was aligned using the MUSCLE algorithm in MEGA 5 with the default parameters. The 12S gene was aligned using the strategy Q_INS-I implemented in MAFFT v6 [32], which takes into account 12S secondary structure [33]. The two mtDNA genes were concatenated in BioEdit 7.0.5.3 [34] and analysed together. All sequences generated during this study are available from GenBank (KM677689-KM677904) (S2 Table).

### Phylogenetic Analysis and Divergence Times

We checked for substitution saturation using the I_SSC_ statistic in the program DAMBE 5.2.27 [35], [36]. We did not find evidence of substitution saturation in 3^rd^ codon position (I_SSC_ < I_SSC, C_), and therefore all positions were used for phylogenetic analysis and estimates of divergence time. The best evolutionary nucleotide model for maximum likelihood (ML) and Bayesian inference was selected using Partition Finder v.1.1.1 [37]. Phylogenetic trees were inferred for the different haplotypes using both ML and Bayesian inference. We conducted the ML analysis in the program RAxML 7.7.1 [38] on the web server http://embnet.vital-it.ch/raxml-bb using the GTR+G model for each of the three different partitions identified by Partition Finder: 12S gene plus 1^st^ codon position of the cyt*b* gene, the 2^nd^ codon position of the cyt*b* gene, and the 3^rd^ codon position of the cyt*b* gene. RAxML uses GTR+G as the default model upon which simpler models can be derived depending on the parameter values that best fit the observed data. Searches were based on 100 rapid bootstrap replicates (RBS), followed by a thorough ML search. RAxML analyses were run in three replicates to check for convergence in tree topology and likelihood score. Bayesian inference was performed using the BEAST 1.8.0 software [39] with nucleotide partitions and models suggested by Partition Finder (HKY+G for 12S+cyt*b*1^st^, HKY+I for cyt*b*2^nd^, and TrN93+G for cyt*b*3^rd^). Analyses based on different haplotypes used a Yule tree-prior and 20 million steps of the Markov Chain Monte Carlo (MCMC) sampling every 1,000 steps. This program was also used to compute the Bayes Factor to compare alternative mtDNA haplotype tree topologies based on a Path Sampling strategy [40]. We assessed chain convergence by comparing the results of at least three independent runs, and we considered MCMC sampling to be sufficient whenever ESS values were above 200 for all parameters. Convergence and the effective sample size of all MCMC runs were checked in Tracer 1.6 (http://tree.bio.ed.ac.uk/software/tracer/). The support values for the clades obtained in the Bayesian analysis are given in terms of their posterior probability (PP), which is conditioned on the trees sampled along the MCMC run. There are no fossils or known geological events for this group that could be used for a specific estimate of the substitution rate. Therefore, we used the evolutionary rate estimated by Arnold *et al*. [41], who analysed the same mtDNA genes for *Hemidactylus*, a gecko from the Gekkonidae family, and suggested an evolutionary rate of 1.15% per million years (Myr^-1^). This rate is consistent with the widely used “general reptile mtDNA rate” that was reported by Zamudio & Greene [42], which ranges from 0.47% Myr^-1^ to 1.32% Myr^-1^. It is also consistent with estimates for other mtDNA genes reported for *Crenadactylus ocellatus*, a gecko from the Diplodactylidae family [43]. Divergence times were estimated in BEAST assuming the strict clock model and a normal distribution for the substitution rate, with a mean of 1.15% Myr^-1^, as explained above, and a standard deviation of 0.20% Myr^-1^ to allow for some uncertainty in the evolutionary rate. The strict clock model is usually well justified when most data are intraspecific or from closely related species [44]. A recent phylogenetic study in *Homonota* failed to reject a strict clock model even with interspecific data and older divergence events compared to our study [19].

### Genetic and Geographic Structure

Summary statistics, such as the number of different haplotypes, haplotype and nucleotide diversity, were estimated in the program Arlequin 3.5 [45]. This program was also used to assess the level of genetic structure among subpopulations using Φ_ST_, which is analogous to Wright’s F-statistics but takes into account the genetic distance among haplotypes [46]. The evolutionary relationship among haplotypes was inferred using a median-joining network [47] in the program Network 4.6 (www.fluxus-engeneering.org). The correlation between genetic and geographic distances was tested using a Mantel test [48] in the program Alleles in Space 1.0 [49].

We tested for deviations from the null hypothesis of a constant population size using the neutrality tests of Tajima’s D [50] and Fu’s F_S_ [51] in the program Arlequin 3.5 [45]. We also performed a Bayesian Skyline plot (BSP) analysis, which does not assume *a priori* any growth model and infers effective population size through time based on coalescent theory [52]. The BSP was estimated in the program BEAST 1.8.0 [39] as described above, except that the coalescent BSP tree-prior was used instead of the Yule model used in the previous analysis. A joint estimate of the effective population size and exponential growth rate for each population was also performed using the Bayesian search strategy in the program LAMARC v. 2.1.6 [53]. The estimate was based on three replicates of 20 short initial chains of 10,000 steps and two long chains of 1 million steps sampled every 100 steps. Initially, we estimated effective population size and population growth simultaneously to test for significant growth. As a next step, for all populations with no evidence of population growth, we estimated the effective population size based on a constant population size model using the search strategy detailed above. This strategy aimed to reduce the uncertainty in population size due to large but uninformative values for the growth parameter. We assumed the same partitions for demographic estimates in BEAST and LAMARC as in the phylogenetic analyses.

### Morphological Analysis

To compare the level of genetic structure and the degree of morphological differentiation, we measured 16 traits in 79 adult individuals (35 male, 44 female) for which we had sequence data. Individuals were considered adults if they were larger than 38.47 mm for males or 35.08 mm for females (L. Martins, personal communication). We measured the following traits using a digital pachymeter with 0.01 mm precision: Snout-vent length (SVL), head length (HL), head width (HW), neck length (NL), neck width (NW), snout length (SL), base tail width (BTW), interlimb length (ILL), humerus length (HUL), forearm length (FAL), tibia length (TL), femur length (FL), length of the third and fourth fingers (3FL and 4FL), and length of third and fourth toes (3TL and 4TL).

Initially, we extracted the natural logarithm of all values and tested the interdependence of traits HL vs. HW, and NL vs. NW using a standard regression. We tested for sexual dimorphism using Student’s t or Mann-Whitney test depending on the normality of data, which was tested using the Shapiro-Wilk W test. Differences in SVL among sampling sites were tested using one-way ANOVA and the post-hoc Tukey test. We used a forward stepwise discriminant analysis to estimate which variables were the most effective in assigning an individual to its original population. All analyses of morphological data were performed in the programs PAST v.2.02 [54] and STATISTICA v.7.1 (StatSoft Inc).

## Results

A total alignment of 1,088 bp, including the two mtDNA genes (694 bp for cyt*b*; 394 bp for 12S), was obtained for all 108 individuals. Overall, we identified 127 variable sites, of which only 15 were singletons, and 50 different haplotypes for the concatenated mtDNA genes in *H*. *uruguayensis*. The alignment of the 12S gene also showed five polymorphic indels in *H*. *uruguayensis*, which were treated as missing data in further analyses. Of these, two may represent synapomorphies (one deletion shared by Clades III and IV, one insertion shared by two haplotypes from Clade IV), and the other three are most likely homoplasies occurring in different branches of the mtDNA haplotype tree. We did not find substitutions causing insertion-deletion polymorphisms or stop codons for the cyt*b* sequences, nor double peaks in the chromatograms that suggested a heterozygous individual. These results suggest that our sequences are genuine mtDNA sequences rather than inserts of mtDNA in the nucleus (NUMTs).

### Phylogenetic Analysis and Time Estimates

**Fig 2:**
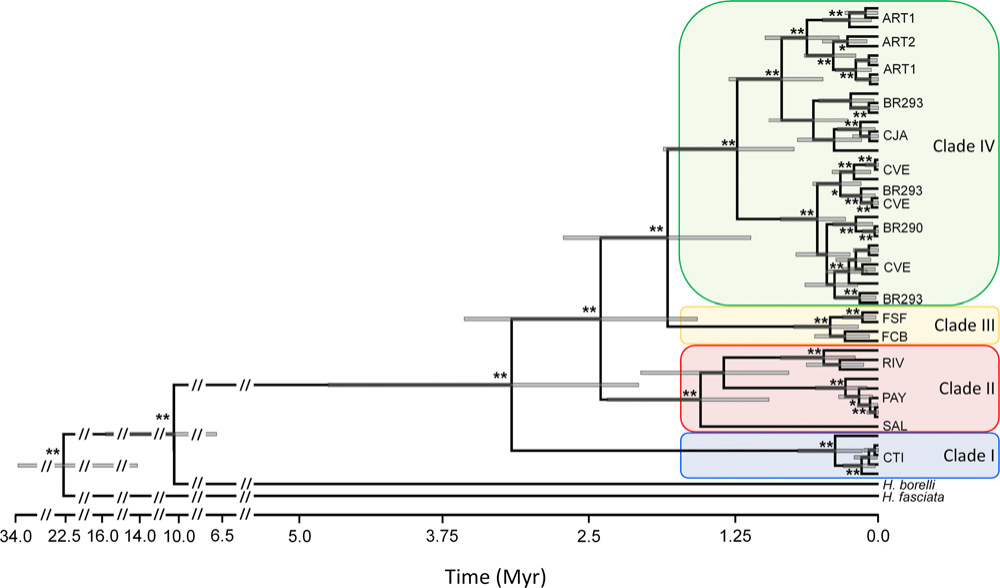
A time-calibrated Bayesian evolutionary tree produced in BEAST (see text for details). **posterior probability (PP) > 0.95; *PP > 0.90. Time is given in millions of years (Myr). The bars for each node represent the 95% credible interval for the time of the most recent ancestor (TMRCA). Clades I-IV are shaded in agreement to Fig. 1.

ML and Bayesian methods resulted in different trees. In the ML tree (S1 Fig.), the *H*. *uruguayensis* clade receives only moderate support (Bootstrap = 84), and haplotypes from ART1 (all populations abbreviations follow those in Table 1) appear paraphyletic to all others. On the other hand, the Bayesian tree (Fig. 2) recovered the *H*. *uruguayensis* clade with maximum support (PP = 1.00) with a clade containing the haplotypes from the CTI sister to all others (PP = 0.99). The long branch between *H*. *uruguayensis* and the closest outgroup (*H*. *borellii*) could suggest an unstable root in the ML analysis. To test whether both topologies were equally likely, we ran a Bayesian analysis enforcing the topology found by the ML search and then compared the analyses using Bayes Factors, which indicated that the unconstrained topology found in our original Bayesian analysis is ~10^37^ times better supported than the alternative ML topology, which is highly significant [55]. Therefore, we based all of our data interpretation on the Bayesian phylogenetic tree.

There are four major mtDNA lineages in *H*. *uruguayensis* (which we call Clade I-IV) that diverged more than 1 Myr ago, and several well supported clades within these four major Clades (which we call “subclades”) (Fig. 2). Clade I is exclusive of the CTI population, Clade II contains the haplotypes for the Uruguayan populations RIV, PAY, and SAL, Clade III contains haplotypes found in FSF and FCB, and Clade IV contains haplotypes found in all remaining populations that are located more “centrally” in the species distribution (see Fig. 1). Only a few populations show evidence of having some phylogenetic structure. For example, BR293 had haplotypes from two different subclades; ART1 was paraphyletic in relation to ART2, and CVE also exhibited some internal structure, though not as much as BR293. Interestingly, all populations with higher phylogenetic structure are located within the central region of the species distribution.

The molecular clock estimates put the divergence between *H*. *fasciata* and *H*. *borellii* in the Early Miocene, approximately 22.5 Myr (with the 95% credible interval (CI) between 14.23 and 33.61 Myr). However, the divergence between *H*. *borellii* and *H*. *uruguayensis* would have occurred in the Late Miocene, approximately 10.51 Myr (95% CI between 6.80 and 15.80 Myr). The estimated coalescence of all *H*. *uruguayensis* mtDNA lineages would have occurred in the Late Pliocene, approximately 3.16 Myr (95% CI between 2.07 and 4.78), with the divergences between clades occurring steadily afterwards (Fig. 2).

### Genetic Structure and Population History

**Fig 3:**
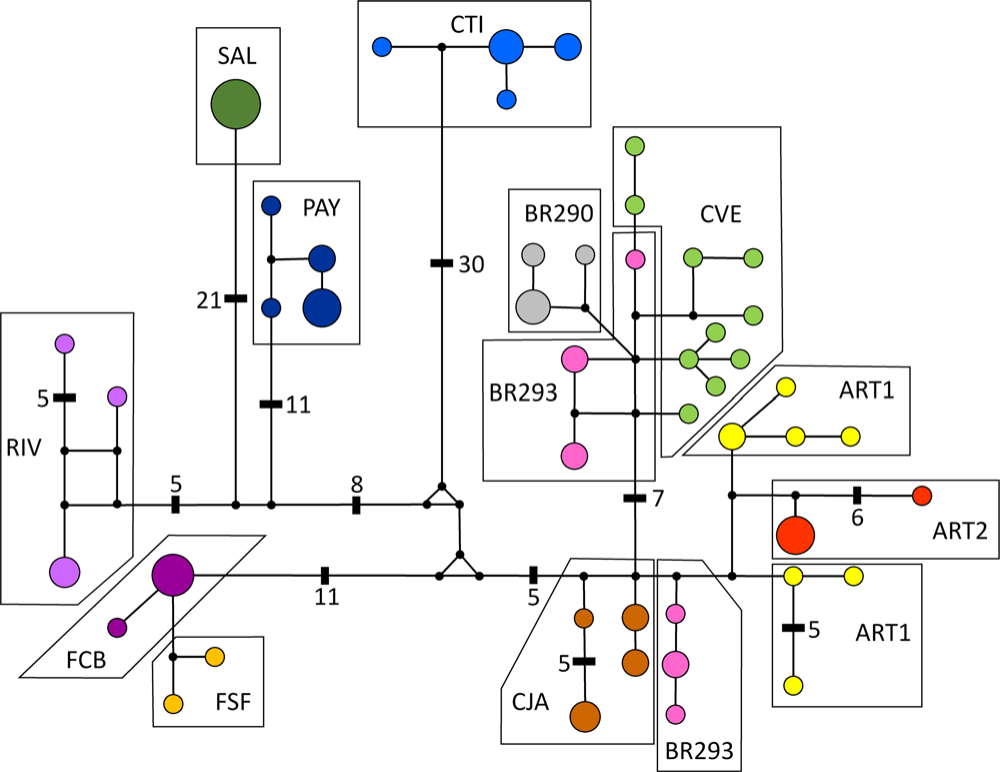
Haplotype network obtained under the median-joining method. Population labels are given according to Table 1. The size of each circle is proportional to haplotype frequency. Median vectors are represented by small back circles. All lines represent one mutational step except when noted.

In general, haplotype diversity was high, with an average of 0.97 for the whole sample, but there were considerable differences among populations (Table 1). We excluded FSF from all population and morphological analyses because we only sampled two individuals from that population. Populations from the central region usually had higher values (CJA, BR293, CVE, ART1), as did the divergent CTI population. On the other hand, populations such as SAL, FBC and ART2 (the latter also from the “central region”) had lower values. The haplotype network (Fig. 3) shows no haplotype sharing among localities, and that 64% of all haplotypes were singletons. This finding reinforced the high haplotype diversity for this species as a whole and the deep divergence among clades. For example, there are at least 40 mutational steps between haplotypes found in CTI (Clade I) to haplotypes from the central region (Clade IV), 25 steps between Clades II and IV and 18 steps between Clades III and IV.

Such strong genetic structure is also clear from the Φ_ST_ values (Table 2), which ranged from 0.24 (CVE vs. BR293) to 0.99 (SAL vs. CTI). Additionally, as expected from the haplotype network, the Φ_ST_ between populations from the central region were generally lower than any other pairwise comparison, including populations from the same clade. Uncorrected pairwise genetic distances ranged from 0.8% (CJA vs. BR293) to 5.4% (SAL vs. CTI), whereas intrapopulational distances ranged from 0.0% (SAL) to 0.7% (BR293) (Table 2). At the mtDNA clade level, the mean distance among clades was 3.7%, ranging from 2.4% (Clade II *vs*. Clade IV) to 4.8% (Clade I vs. Clade II), whereas distances within clades ranged from 0.3% (Clade I) to 1.4% (Clade II). The Mantel test showed that geographic distance was significantly associated with genetic distance (P = 0.0009) and accounted for approximately 40% of the variance in genetic distances (r^2^ = 0.394).

**Table 2:** Genetic structure among ***H*. *uruguayensis*** and outgroups.

Population	1	2	3	4	5	6	7	8	9	10	11	12	13
1 CJA	**0.003**	0.480	0.873	0.849	0.939	0.688	0.581	0.781	0.869	0.925	0.954	-	-
2 BR293	0.008	**0.007**	0.798	0.513	0.897	0.243	0.528	0.692	0.799	0.874	0.911	-	-
3 FCB	0.021	0.022	**0.005**	0.936	0.957	0.841	0.837	0.924	0.917	0.953	0.972	-	-
4 BR290	0.013	0.009	0.025	**0.002**	0.971	0.514	0.796	0.934	0.937	0.969	0.990	-	-
5 CTI	0.038	0.039	0.042	0.042	**0.003**	0.920	0.905	0.961	0.945	0.970	0.986	-	-
6 CVE	0.013	0.008	0.023	0.006	0.040	**0.004**	0.667	0.800	0.847	0.909	0.938	-	-
7 ART1	0.010	0.012	0.026	0.017	0.040	0.017	**0.006**	0.438	0.833	0.898	0.926	-	-
8 ART2	0.013	0.014	0.027	0.020	0.040	0.019	0.009	**0.006**	0.921	0.961	0.983	-	-
9 RIV	0.026	0.027	0.033	0.031	0.044	0.030	0.032	0.034	**0.006**	0.889	0.947	-	-
10 PAY	0.029	0.030	0.035	0.034	0.047	0.033	0.035	0.038	0.020	**0.002**	0.978	-	-
11 SAL	0.035	0.037	0.039	0.042	0.054	0.040	0.041	0.042	0.027	0.029	**0.000**	-	-
12 *H*. *fasciata*	0.164	0.166	0.168	0.169	0.174	0.167	0.162	0.161	0.173	0.169	0.171	-	-
13 *H*. *borellii*	0.106	0.109	0.113	0.109	0.115	0.111	0.108	0.107	0.112	0.113	0.120	0.170	-

Above diagonal—Pairwise Φ_ST_ values; Diagonal (in bold)—Intrapopulational pairwise distances; Below diagonal—Pairwise genetic distances.

**Fig 4:**
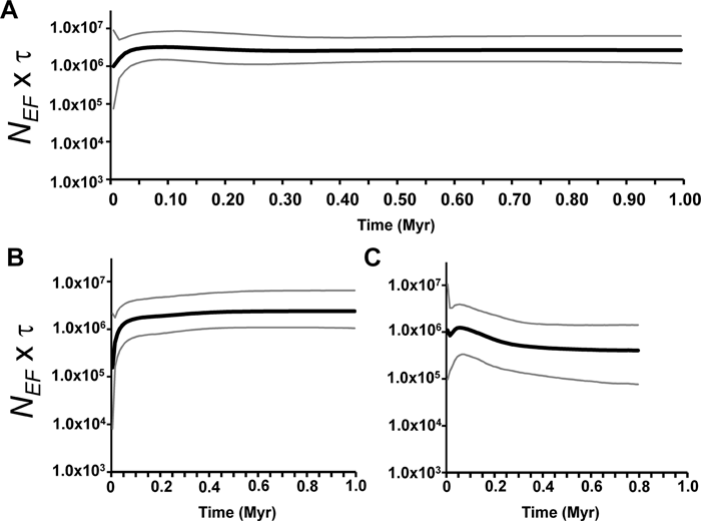
Bayesian Skyline Plot. A—All populations; B—Peripheral populations; C—Clade IV (see text for details). The x-axis is given in millions of years (Myr). The y-axis is given in Effective female population size (*N*_*EF*_) multiplied by generation time (τ).

At the population level, demographic estimates from LAMARC showed evidence of population growth only for CVE; at the clade level, there was evidence of population growth only for Clade IV. There was no evidence of population growth for the whole species or for Clades I-III combined (“Peripheral populations”; Table 3), suggesting that population growth in Clade IV is not an artefact of analysing more samples. It is also noteworthy that some populations from Clade IV (CVE, BR290, ART2) produced significant results in one of the neutrality tests (Table 1). In agreement with these results, the BSP for the entire species showed a pattern of constant population size (Fig. 4A). Comparing the BSP dynamics for the Peripheral populations and Clade IV, we found no signal of a population expansion in the former population (Fig. 4B). However, the data suggest that Clade IV underwent a population expansion beginning approximately 250,000 years ago, even though credible intervals are broad (Fig. 4C). The signals of a likely population reduction in recent times are most likely artefacts of using pooled samples from structured populations, which mimics the coalescence behaviour of lineages from a shrinking population [56]. Taking into account the estimate of five years as the generation time in *H*. *darwinii* [57], the effective female population size of *H*. *uruguayensis* is approximately 350,000 individuals (Table 3). Estimates from BSP and LAMARC are in general agreement, even though the credible intervals for BSPs are very large. Population size estimates for CVE, in the presence of population growth, resulted in unrealistic large values. Thus, we have analysed the CVE population under the constant size model to set a lower bound for this parameter. Even in the constant population model, CVE has the largest effective female population size (Table 3), followed by ART1 and BR293, all belonging to Clade IV.

**Table 3:** Estimates of Female Effective Population Size (N_EF_) and population growth.

Population	Theta	CI 95%	Mean N_EF_	CI 95%	Growth^1^	CI 95%
ART1	5.77x10^–3^	2.43x10^–3^–1.58x10^–2^	50,157	21,104–136,974	NS	NS
ART2	1.62x10^–3^	4.56x10^–4^–5.34x10^–3^	14,043	3,965–46,426	NS	NS
BR290	8.81x10^–4^	1.41x10^–4^–3.14x10^–3^	7,661	1,226–27,270	NS	NS
BR293	4.51x10^–3^	1.90x10^–3^–1.09x10^–2^	39,174	16,513–94,870	NS	NS
CJA	1.90x10^–3^	6.13x10^–4^–5.65x10^–3^	16,530	5,330–49,157	NS	NS
CTI	2.06x10^–3^	6.46x10^–4^–6.30x10^–3^	17,922	5,617–54,739	NS	NS
CVE^2^	5.16x10^–2^	8.84x10^–3^–2.94x10^0^	448,965	76,852–23,454,843	1613	408–6063
CVE^3^	7.71x10^–3^	3.34x10^–3^–1.98x10^–2^	67,070	29,070–172,026	0*	0*
PAY	1.78x10^–3^	4.62x10^–4^–5.32x10^–3^	15,461	4,017–46,217	NS	NS
RIV	4.08x10^–3^	1.40x10^–3^–1.45x10^–2^	35,443	12,191–126,113	NS	NS
FCB	1.01x10^–3^	2.25x10^–4^–3.53x10^–3^	8,748	1,957–30,704	NS	NS
SAL	1.57x10^–5^	1.01x10^–5^–6.98x10^–4^	137	88–6,070	NS	NS
Clade I	2.06x10^–3^	6.46x10^–4^–6.30x10^–3^	17,922	5,617–54,739	NS	NS
Clade II	9.05x10^–3^	5.44x10^–3^–1.61x10^–2^	78,730	47,339–140,191	NS	NS
Clade III	3.12x10^–3^	1.22x10^–3^–8.45x10^–3^	27,165	10,574–73,496	NS	NS
Clade IV	2.05x10^–2^	1.38x10^–2^–3.02x10^–2^	178,026	119,626–262,296	283	33–315
“Peripheral” Populations^4^	1.95x10^–2^	1.37x10^–2^–2.96x10^–2^	169,487	119,148–257,148	NS	NS
All Populations	3.89x10^–2^	3.09x10^–2^–5.17x10^–2^	337,904	268,452–449,235	NS	NS

CI 95%- 95% Credible interval for the estimates.

^1^Prior bounds for population growth between-5,000 and 15,000.

^2^Estimates for female effective population size assuming exponential population growth.

^3^Estimates for female effective population size assuming constant population size.

^4^”Peripheral” populations include Clades I, II and III. NS—Not significant.

*See text for details.

### Morphological Analysis

All of the raw morphological measures are available in S1 Table. No correlations were found between HL and HW or NL and NW (P>0.05), and therefore all of these traits were included in further analysis. Males and females were separately analysed for the six (HL, HW, NL, NW, 3TL, 4TL) out of 16 traits that differed significantly between sexes (P<0.05). For population comparisons, the RIV population was excluded from the analysis of males because only a single male individual was sampled in this population. The stepwise discriminant analysis of females identified eight steps (Wilks’ Lambda = 0.0069, F(80,173) = 2.5833, P<0.0001), for which the traits NL, SL, ILL, NW, and HUL were the most important, whereas 12 steps were identified for males (Wilks’ Lambda = 0.0018, F(108,143) = 1.8188, P = 0.0004), for which the most important traits were HL, FL, NL, and 4TL. Overall, in spite of the low sample size for each population, 82% of both males and females were correctly assigned to their original population, and populations CTI, CJA and ART2 had 100% correct assignments for both sexes. These results indicate that there are morphological differences among populations in this species.

## Discussion

### Genetic Structure and Polymorphism

Our data reveal a strong genetic structure in *H*. *uruguayensis*, for which mtDNA haplotypes show a deep evolutionary history. The genetic distances between *H*. *uruguayensis* clades are comparable to those found between different species of the Lacertidae family, which has 2.5 to 6.4% interspecific distances for the mtDNA 12S gene [58]. Even when the comparison involves other geckos (Phyllodactylidae [59] and Gekkonidae [60]-[62]), the values observed in this study are still high. In the genus *Tarentola*, values for the Kimura 2-parameter distance between subspecies and species ranged from 3 to 9% for the same genes used in our study [30]. Applying this genetic distance to our data produces a 6.5% divergence between Clade I and Clade II and an approximate 5% divergence between Clade I and either Clade III or IV. Deep divergences have also been reported for some phyllodactylid geckos. For example, there are at least eight deep evolutionary lineages that may represent different species in the *Phyllopezus pollicaris* complex [63]. However, *P*. *pollicaris* is distributed across the whole system of open South American biomes known as the “dry diagonal” that spans from northeastern Brazil to northeastern Argentina, which complicates the comparison with a species of restricted distribution like *H*. *uruguayensis*. Nonetheless, the results found in our study, *P*. *pollicaris* and other non-Phyllodactylidae South American gecko complexes [26] indicate that a strong mtDNA structure may be a common pattern for these organisms.

The strong genetic structure found for mtDNA (Fig. 2, Table 2) might reflect a difference in female/male dispersal rather than a general trend for this species. It is known that several species exhibit male-biased dispersal, which would result in high values of mtDNA structure but lower genome-wide levels (e.g., [64]). However, previous field work on this species suggests that this is not the case. Vieira [25] tracked individuals from the FCB population and found that both home range and movement patterns were higher for females than males, both in the breeding and non-breeding seasons. In any case, dispersal was very limited, as the maximum dispersal recorded in the non-breeding season was 182.52 m for a female and 81.83 m for a male. In the breeding season, these values were 190.38 m for a female and 33.61 m for a male. Such strong philopatry for both sexes is most likely associated with the high habitat specificity of *H*. *uruguayensis*, which is found exclusively in basalt outcrops used for shelter and nesting [18].

### Phylogeographic Hypotheses and Evolutionary History

The evolutionary origin and initial diversification of *Homonota* dates back to the Early Miocene, based on an estimated 20 to 15 My origin of *Homonota* [19], [65]. This time period coincides with the initial phase of the rise of the Andes [66], which affected many groups of South American animals (e.g., [67]). Another important factor affecting southern South America were the three marine transgressions in the Middle to Late Miocene, which ultimately led to the formation of wide plains that were rapidly colonised by grasslands when the sea level decreased [68]. These processes were most likely important in the evolutionary history of *Homonota*, as they first extinguished ancestral lineages and then allowed the diversification of new lineages from ancient “refuge” areas unaffected by marine transgressions [19], [69]. Indeed, the origin of *H*. *uruguayensis* itself may be related to a marine transgression event [19].

Three major rivers in the Brazilian and Uruguayan Pampas (the Uruguay river, to the west, the Negro river to the south, and the Ibicuí river to the North) set the bounds of the species distribution, which is predicted for species with low dispersal abilities [26], [70]. These rivers may act as geographical barriers for other species. For example, it has been suggested that the Ibicuí river is associated with genetic isolation between species and populations of *Ctenomys*, a subterranean mammal [71], [72], and that the Negro river may act as a barrier for two subspecies of the plant *Petunia axillaris* [15].

Our results may be reconciled with a historical scenario in which individuals from a centrally distributed meta-population eventually disperse to colonise new areas, or a scenario in which a more highly connected population eventually loses connectivity with its peripheral populations and thus strengthens its genetic structure. We consider the second hypothesis more likely because, in the first scenario, recurrent dispersal from the same “source” population would have resulted in paraphyly of the genetic lineages in this population, as is the case for recent island species of *Bothrops* vipers [73]. Nevertheless, in both cases, the most important geographic feature explaining genetic and population divergence may be rivers, which may have varied in course, water flow, or riparian vegetation through time. The extension of gallery forest along the river margins appears to have changed between glacial and interglacial cycles [6].This extension might have facilitated population isolation during periods of forest expansion, at which time suitable rocky outcrops would not be available for *H*. *uruguayensis*.

In general, the distribution of mtDNA clades is concordant with the distribution of major rivers (Fig. 1). The two possible exceptions to this rule occur in Clade IV. The Ibirapuitã river, which may be a barrier separating Clades III and IV downstream, does not act as an effective barrier upstream, as populations CVE and BR293 are closely related, even though there is no haplotype sharing between them. Our results also failed to suggest that the Quaraí river is an historic barrier for *H*. *uruguayensis*, because Clade IV is distributed on both banks of this river (Fig. 1). Although the ART1 and ART2 populations (in the Uruguayan bank) form a well-supported subclade within Clade IV, other populations from the Brazilian side show comparable values of genetic structure (Table 3). These examples may suggest that geographic distance per se may provide a more parsimonious explanation for our results, as geographic distances accounted for approximately 40% of the genetic distances in the Mantel test. Alternatively, these rivers may be better seen as an unstable barrier, allowing population connection during certain periods, but restricting gene flow on other occasions. A similar mechanism (i.e., fluctuation in flow volume) has been evoked to explain the genetic structure of lizards [74], [75] and rodents [76] across the São Francisco River, in northeastern Brazil.

The stringent ecological requirements of *H*. *uruguayensis* may explain why this species has a limited distribution despite the lack of obvious geographic barriers to the east of the species distribution. Together with the low dispersal abilities of *H*. *uruguayensis*, such stringency may have facilitated population isolation. Indeed, the basalt outcrops used for shelter and nest construction by this species may be considered ecological “islands” in the grassland “ocean” of the Pampas. Because of its low dispersal, this species is highly dependent on stepping-stones of suitable areas to connect different populations in order to maintain genetic connectivity among populations, colonise new habitats, and re-colonise areas after local extinction. Therefore, despite rivers representing reasonable barriers to gene flow, the strong population isolation within each clade may simply reflect low mobility coupled with high ecological specificity of *H*. *uruguayensis*.

Genetic diversity values and demographic estimates suggest important differences in the demographic dynamics between different regions across the species distribution (Table 1, Table 3, Fig. 4). In general, populations from the central part of the distribution are more variable and have larger female effective population sizes, although the credible intervals are broad because only mtDNA was used in this analysis [77]. The population expansion that affected populations from the central part of the species distribution is roughly coincident with the origin of several mtDNA subclades for Clade IV (Fig. 2, Fig. 4). Given that there is no haplotype sharing and that all subclades are restricted to a single geographic population, it is possible to infer that several rocky outcrops in the central region were colonised (or re-colonised) during this expansion, with genetic drift and population isolation later restricting the number of subclades in each population. Turchetto *et al*. [15] also documented a population expansion for *Petunia axillaris parodii* that is sympatric to *H*. *uruguayensis*. However, the population expansion for *P*. *a*. *parodii* is more recent, around 0.1 Myr and therefore not driven by the same environmental conditions. To our knowledge, all other studies of Pampean species tend to suggest dates more recent than 0.1 Myr, either for population expansion or divergence among genetic lineages [13]-[16]. Thus, it is striking that we have estimated a population expansion in a single clade older than 0.1 Myr. These results highlight that even in a biome mostly covered by grasslands during the entire Pleistocene, demographic patterns exhibited by different species may be largely discordant.

On the other hand, common evolutionary patterns among different species may also exist. Speranza *et al*. [14] found high chloroplast DNA diversity for populations of the plant *Turnera sidoides* occurring in the Haedo Range, which is a range of hills less than 500 m in height located in the north-northwest Uruguay. These authors also suggest that this area could have served as a refuge for *T*. *sidoides*, even though they did not analyse plants from Brazil, where the species also occurs. For *H*. *uruguayensis*, two populations with high nucleotide diversity (Table 1) and effective female population sizes (Table 3) were CVE and BR293, which are located close to the “Coxilha Grande”, the continuation of the Haedo Range in Brazil. In addition, RIV in the Uruguayan portion of the Haedo Range also has high genetic diversity. This observation may support the role of the Haedo Range as a region of high genetic diversity for several Pampean species.

### Morphology and Conservation

There are also morphological differences among populations, with 82% of all individuals being correctly assigned to their original population based on morphological data alone. For some populations, all individuals were correctly assigned. As the ecological context for all populations is similar, the strong population structure for *H*. *uruguayensis* may suggest that the morphological differences among populations are due to genetic drift [78]. However, it is controversial that genetic drift may be strong enough to result in morphological change in natural populations [79]-[83], and therefore we cannot exclude the role of local adaptive processes affecting *H*. *uruguayensis* morphology. It is possible that the CTI population, which has the most divergent mtDNA clade and 100% correct morphological identifications, reflects an evolutionary lineage distinct enough to be considered a different species or subspecies. However, a more comprehensive morphological characterisation and the study of variable nuclear genetic loci are necessary to answer this question. Finally, another implication of the deep population structure is that each local population contains unique genetic variation, and therefore can be considered at least as independent management units (MU) *sensu* Moritz [84], which is important for conservation plans for *H*. *uruguayensis*.

## Supporting Information

S1 Fig Maximum likelihood tree for *H*. *uruguayensis* mtDNA haplotypes. Note the low bootstrap support values (values below 70 were omitted), and the alternative topology showing haplotypes from ART1 as sister to all other *H*. *uruguayensis* haplotypes. This topology was strongly rejected based on the Bayes Factor in favour of a topology where haplotypes from CTI are sister to all others (see text for details).
(DOC)

S1 Table Morphological measures (Mean ± Standard Deviation) for *H*. *uruguayensis* populations. (DOC)

S2 Table Genbank accession numbers for all individuals analysed in this study. Voucher numbers in scientific collections can be obtained from the respective Genbank files.
(DOC)
