# Supplementary material for: So Far Away, Yet So Close: Strong Genetic Structure in Homonota uruguayensis (Squamata, Phyllodactylidae), a Species with Restricted Geographic Distribution in the Brazilian and Uruguayan Pampas
Source: PLoS One. 2015 Feb 18;10(2):e0118162. doi: 10.1371/journal.pone.0118162 (PMC4334718; doi:10.1371/journal.pone.0118162)
Supplement: S1 Fig — Note the low bootstrap support values (values below 70 were omitted), and the alternative topology showing haplotypes from ART1 as sister to all other H. uruguayensis haplotypes. This topology was strongly rejected based on the Bayes Factor in favour of a topology where haplotypes from CTI are sister to all others (see text for details). (DOC) [file pone.0118162.s001.doc]

**Figure S1. Maximum likelihood tree for *H. uruguayensis* mtDNA haplotypes.** Note the low bootstrap support values (values below 70 were omitted), and the alternative topology showing haplotypes from ART1 as sister to all other *H. uruguayensis* haplotypes. This topology was strongly rejected based on the Bayes Factor in favour of a topology where haplotypes from CTI are sister to all others (see text for details).
