# Supplementary material for: So Far Away, Yet So Close: Strong Genetic Structure in Homonota uruguayensis (Squamata, Phyllodactylidae), a Species with Restricted Geographic Distribution in the Brazilian and Uruguayan Pampas
Source: PLoS One. 2015 Feb 18;10(2):e0118162. doi: 10.1371/journal.pone.0118162 (PMC4334718; doi:10.1371/journal.pone.0118162)
Supplement: S1 Table — (DOC) [file pone.0118162.s002.doc]

**Table S1. Morphological measures (Mean ± Standard Deviation) for *H. uruguayensis* populations.**

| Trait^1^ |  | ART1 | |  | ART2 | |  | BR290 | |  | BR293 | |  | CJA | |
| --- | --- | --- | --- | --- | --- | --- | --- | --- | --- | --- | --- | --- | --- | --- | --- |
|  |  | M (N=5) | F (N=3) |  | M (N=3) | F (N=7) |  | M (N=6) | F (N=4) |  | M (N=2) | F (N=3) |  | M (N=3) | F (N=6) |
| SVL |  | 40.75 ± 2.21 | 40.14 ± 1.56 |  | 41.58 ± 2.63 | 39.40 ± 2.93 |  | 41.40 ± 1.47 | 40.49 ± 1.52 |  | 39.94 ± 2.03 | 40.79 ± 1.38 |  | 42.93 ± 1.50 | 40.29 ± 1.84 |
| HL |  | 11.54 ± 0.50 | 10.98 ± 0.17 |  | 12.31 ± 1.07 | 11.12 ± 0.53 |  | 11.68 ± 1.01 | 11.4 ± 0.48 |  | 12.84 ± 0.44 | 11.48 ± 0.70 |  | 11.98 ± 0.73 | 11.21 ± 0.40 |
| HW |  | 7.87 ± 0.25 | 7.77 ± 0.12 |  | 8.03 ± 0.41 | 7.64 ± 0.57 |  | 8.26 ± 0.34 | 7.85 ± 0.50 |  | 7.95 ± 0.42 | 7.73 ± 0.10 |  | 8.44 ± 0.32 | 7.89 ± 0.32 |
| NL |  | 2.04 ± 0.67 | 2.35 ± 0.25 |  | 2.31 ± 0.12 | 2.43 ± 0.60 |  | 2.79 ± 0.80 | 3.61 ± 0.41 |  | 2.06 ± 0.82 | 2.38 ± 0.78 |  | 3.91 ± 0.74 | 4.05 ± 0.48 |
| NW |  | 6.82 ± 0.55 | 7.65 ± 0.67 |  | 7.20 ± 0.67 | 7.17 ± 0.76 |  | 6.88 ± 0.63 | 7.73 ± 0.45 |  | 7.00 ± 0.23 | 7.82 ± 0.71 |  | 7.25 ± 0.08 | 7.21 ± 0.46 |
| SL |  | 3.68 ± 0.17 | 3.70 ± 0.10 |  | 3.65 ± 0.17 | 3.43 ± 0.29 |  | 3.75 ± 0.17 | 3.57 ± 0.13 |  | 3.66 ± 0.20 | 3.67 ± 0.23 |  | 3.95 ± 0.08 | 3.68 ± 0.08 |
| BTW |  | 4.53 ± 0.38 | 4.49 ± 0.16 |  | 4.11 ± 0.40 | 4.20 ± 0.49 |  | 4.54 ± 0.43 | 4.64 ± 0.68 |  | 4.38 ± 0.19 | 4.47 ± 0.20 |  | 4.77 ± 0.27 | 4.31 ± 0.28 |
| ILL |  | 17.34 ± 0.77 | 18.25 ± 0.90 |  | 18.56 ± 2.08 | 17.61 ± 1.75 |  | 17.03 ± 0.77 | 16.51 ± 1.41 |  | 17.31 ± 1.21 | 18.06 ± 0.61 |  | 18.25 ± 0.97 | 17.78 ± 0.85 |
| HUL |  | 3.04 ± 0.68 | 3.62 ± 0.20 |  | 3.35 ± 0.32 | 2.86 ± 0.20 |  | 3.25 ± 0.44 | 3.24 ± 0.56 |  | 2.82 ± 0.27 | 2.71 ± 0.08 |  | 3.38 ± 0.02 | 3.03 ± 0.43 |
| FAL |  | 4.15 ± 0.19 | 4.08 ± 0.39 |  | 4.20 ± 0.29 | 3.79 ± 0.16 |  | 4.11 ± 0.37 | 4.18 ± 0.41 |  | 3.93 ± 0.20 | 3.98 ± 0.21 |  | 4.36 ± 0.11 | 4.25 ± 0.27 |
| TL |  | 4.14 ± 0.33 | 4.04 ± 0.51 |  | 4.25 ± 0.30 | 3.81 ± 0.43 |  | 4.00 ± 0.30 | 4.05 ± 0.19 |  | 4.04 ± 0.20 | 4.15 ± 0.33 |  | 4.48 ± 0.65 | 4.41 ± 0.53 |
| FL |  | 5.39 ± 0.27 | 5.59 ± 0.10 |  | 5.57 ± 0.45 | 5.36 ± 0.58 |  | 5.15 ± 0.33 | 5.09 ± 0.05 |  | 5.41 ± 1.02 | 5.10 ± 0.38 |  | 5.53 ± 0.38 | 5.05 ± 0.50 |
| 3FL |  | 3.50 ± 0.32 | 3.25 ± 0.17 |  | 3.35 ± 0.33 | 3.19 ± 0.32 |  | 3.44 ± 0.23 | 3.32 ± 0.15 |  | 3.17 ± 0.01 | 3.32 ± 0.23 |  | 3.67 ± 0.11 | 3.53 ± 0.23 |
| 3TL |  | 4.20 ± 0.41 | 4.16 ± 0.37 |  | 4.46 ± 0.12 | 3.86 ± 0.41 |  | 4.33 ± 0.21 | 4.25 ± 0.15 |  | 4.16 ± 0.39 | 4.11 ± 0.34 |  | 4.69 ± 0.04 | 4.22 ± 0.22 |
| 4FL |  | 3.59 ± 0.40 | 3.43 ± 0.20 |  | 3.47 ± 0.43 | 3.15 ± 0.28 |  | 3.60 ± 0.22 | 3.40 ± 0.19 |  | 3.34 ± 0.50 | 3.43 ± 0.03 |  | 3.76 ± 0.34 | 3.58 ± 0.04 |
| 4TL |  | 4.79 ± 0.52 | 4.57 ± 0.36 |  | 4.77 ± 0.05 | 4.24 ± 0.51 |  | 4.83 ± 0.29 | 4.68 ± 0.28 |  | 4.67 ± 0.13 | 4.89 ± 0.56 |  | 5.27 ± 0.07 | 4.73 ± 0.56 |

^1^See text for details. M – Males; F – Females; N – sample size.

**Table** S1. Continued.

| Trait^1^ | CTI | |  | CVE | |  | FCB | |  | PAY | |  | RIV | |  | SAL | |
| --- | --- | --- | --- | --- | --- | --- | --- | --- | --- | --- | --- | --- | --- | --- | --- | --- | --- |
|  | M  (N=4) | F  (N=5) |  | M  (N=4) | F  (N=3) |  | M  (N=5) | F  (N=5) |  | M  (N=5) | F  (N=4) |  | M  (N=1) | F  (N=2) |  | M  (N=2) | F  (N=2) |
| SVL | 44.03 ± 1.95 | 41.23 ± 4.20 |  | 38.97 ± 0.18 | 40.95 ± 0.91 |  | 42.35 ± 2.17 | 40.50 ± 2.17 |  | 39.34 ± 1.25 | 40.06 ± 1.91 |  | 40.43 | 39.1 ± 2.59 |  | 40.95 ± 2.32 | 42.17 ± 4.09 |
| HL | 12.82 ± 0.79 | 12.02 ± 0.82 |  | 11.04 ± 0.18 | 11.99 ± 0.91 |  | 12.33 ± 0.86 | 11.62 ± 0.45 |  | 11.12 ± 0.64 | 11.22 ± 0.86 |  | 10.97 | 10.47 ± 1.24 |  | 11.54 ± 0.32 | 11.26 ± 0.18 |
| HW | 8.49 ± 0.38 | 8.28 ± 0.74 |  | 7.57 ± 0.16 | 7.84 ± 0.10 |  | 8.57 ± 0.63 | 8.03 ± 0.37 |  | 7.69 ± 0.55 | 7.54 ± 0.39 |  | 7.71 | 7.68 ± 0.92 |  | 8.49 ± 0.28 | 8.49 ± 0.95 |
| NL | 3.28 ± 0.50 | 3.72 ± 0.71 |  | 2.68 ± 0.71 | 2.92 ± 0.58 |  | 2.22 ± 0.33 | 2.85 ± 0.32 |  | 2.44 ± 0.29 | 2.53 ± 0.44 |  | 2.56 | 2.38 ± 0.16 |  | 2.59 ± 0.17 | 3.36 ± 0.71 |
| NW | 6.96 ± 0.75 | 7.04 ± 1.31 |  | 5.96 ± 0.70 | 6.96 ± 0.92 |  | 7.60 ± 0.60 | 7.90 ± 0.57 |  | 6.60 ± 0.92 | 7.16 ± 0.87 |  | 6.28 | 7.22 ± 1.02 |  | 7.28 ± 0.51 | 8.23 ± 1.21 |
| SL | 4.52 ± 0.17 | 4.28 ± 0.44 |  | 3.49 ± 0.10 | 3.88 ± 0.18 |  | 4.02 ± 0.26 | 3.85 ± 0.18 |  | 3.73 ± 0.24 | 3.58 ± 0.12 |  | 3.55 | 3.65 ± 0.43 |  | 3.59 ± 0.04 | 3.88 ± 0.41 |
| BTW | 5.09 ± 0.27 | 4.31 ± 0.28 |  | 4.40 ± 0.40 | 4.47 ± 0.07 |  | 4.77 ± 0.39 | 4.48 ± 0.32 |  | 4.32 ± 0.37 | 4.32 ± 0.35 |  | 4.63 | 4.23 ± 0.69 |  | 4.63 ± 0.16 | 5.05 ± 0.68 |
| ILL | 18.41 ± 0.87 | 16.95 ± 2.52 |  | 17.19 ± 1.05 | 17.26 ± 0.62 |  | 18.32 ± 1.25 | 17.56 ± 1.53 |  | 16.73 ± 1.35 | 18.08 ± 1.34 |  | 18.15 | 17.69 ± 1.28 |  | 17.30 ± 1.02 | 17.54 ± 2.69 |
| HUL | 3.51 ± 0.58 | 3.72 ± 0.66 |  | 3.11 ± 0.26 | 3.19 ± 0.40 |  | 2.94 ± 0.43 | 3.63 ± 0.33 |  | 2.86 ± 0.43 | 2.86 ± 0.33 |  | 3.22 | 3.38 ± 0.10 |  | 2.87 ± 0.59 | 3.31 ± 1.14 |
| FAL | 4.85 ± 0.25 | 4.53 ± 0.55 |  | 4.06 ± 0.08 | 4.12 ± 0.08 |  | 4.14 ± 0.22 | 4.24 ± 0.42 |  | 3.77 ± 0.22 | 3.78 ± 0.15 |  | 3.84 | 4.08 ± 0.05 |  | 4.37 ± 0.17 | 4.41 ± 0.34 |
| TL | 5.43 ± 0.47 | 4.93 ± 0.47 |  | 3.87 ± 0.40 | 4.02 ± 0.29 |  | 4.08 ± 0.27 | 4.36 ± 0.17 |  | 3.94 ± 0.34 | 3.95 ± 0.40 |  | 4.43 | 3.93 ± 0.46 |  | 4.50 ± 0.13 | 4.64 ± 0.71 |
| FL | 6.64 ± 0.38 | 5.75 ± 0.71 |  | 5.07 ± 0.35 | 5.48 ± 0.36 |  | 5.31 ± 0.47 | 5.45 ± 0.42 |  | 5.60 ± 0.51 | 5.20 ± 0.37 |  | 5.51 | 5.21 ± 0.42 |  | 5.86 ± 0.15 | 5.45 ± 0.98 |
| 3FL | 3.75 ± 0.38 | 3.66 ± 0.22 |  | 3.44 ± 0.51 | 3.49 ± 0.24 |  | 3.31 ± 0.44 | 3.49 ± 0.37 |  | 3.41 ± 0.06 | 3.22 ± 0.26 |  | 3.00 | 3.32 ± 0.11 |  | 3.78 ± 0.02 | 3.66 ± 0.47 |
| 3TL | 4.90 ±  0.35 | 4.72 ± 0.49 |  | 4.39 ± 0.49 | 4.37 ± 0.05 |  | 4.32 ± 0.30 | 4.00 ± 0.27 |  | 4.07 ± 0.26 | 4.03 ± 0.19 |  | 4.05 | 4.16 ± 0.14 |  | 4.95 ± 0.05 | 4.53 ± 0.33 |
| 4FL | 4.03 ± 0.25 | 3.89 ± 0.24 |  | 3.50 ± 0.53 | 3.59 ± 0.37 |  | 3.32 ± 0.49 | 3.56 ± 0.24 |  | 3.43 ± 0.11 | 3.39 ± 0.39 |  | 3.02 | 3.34 ± 0.05 |  | 3.78 ± 0.36 | 3.73 ± 0.40 |
| 4TL | 5.21 ± 0.48 | 4.89 ± 0.53 |  | 4.56 ± 0.46 | 4.85 ± 0.36 |  | 4.83 ± 0.23 | 4.64 ± 0.36 |  | 4.48 ± 0.33 | 4.41 ± 0.24 |  | 4.93 | 4.23 ± 0.67 |  | 5.11 ± 0.08 | 4.96 ± 0.20 |

^1^See text for details. M – Males; F – Females; N – sample size.
