# Supplementary material for: So Far Away, Yet So Close: Strong Genetic Structure in Homonota uruguayensis (Squamata, Phyllodactylidae), a Species with Restricted Geographic Distribution in the Brazilian and Uruguayan Pampas
Source: PLoS One. 2015 Feb 18;10(2):e0118162. doi: 10.1371/journal.pone.0118162 (PMC4334718; doi:10.1371/journal.pone.0118162)
Supplement: S2 Table — Voucher numbers in scientific collections can be obtained from the respective Genbank files. (DOC) [file pone.0118162.s003.doc]

Table S2. Genbank accession numbers for all individuals analysed in this study. Voucher numbers in scientific collections can be obtained from the respective Genbank files.

| Individual | Population | Cyt*b* | 12S |
| --- | --- | --- | --- |
| Jarau_2241 | CJA | KM677689 | KM677797 |
| Jarau_2242 | CJA | KM677690 | KM677798 |
| Jarau_2243 | CJA | KM677691 | KM677799 |
| Jarau_2244 | CJA | KM677692 | KM677800 |
| Jarau_2245 | CJA | KM677693 | KM677801 |
| Jarau_2246 | CJA | KM677694 | KM677802 |
| Jarau_2247 | CJA | KM677695 | KM677803 |
| Jarau_2248 | CJA | KM677696 | KM677804 |
| Jarau_2249 | CJA | KM677697 | KM677805 |
| Jarau_2559 | CJA | KM677698 | KM677806 |
| BR293_2169 | BR293 | KM677699 | KM677807 |
| BR293_2170 | BR294 | KM677700 | KM677808 |
| BR293_2171 | BR295 | KM677701 | KM677809 |
| BR293_2172 | BR296 | KM677702 | KM677810 |
| BR293_2173 | BR297 | KM677703 | KM677811 |
| BR293_2175 | BR298 | KM677704 | KM677812 |
| BR293_2176 | BR299 | KM677705 | KM677813 |
| BR293_2177 | BR300 | KM677706 | KM677814 |
| BR293_2178 | BR301 | KM677707 | KM677815 |
| BR293_2179 | BR302 | KM677708 | KM677816 |
| BR293_2180 | BR303 | KM677709 | KM677817 |
| Rosario_1311 | FCB | KM677710 | KM677818 |
| Rosario_1312 | FCB | KM677711 | KM677819 |
| Rosario_1313 | FCB | KM677712 | KM677820 |
| Rosario_1315 | FCB | KM677713 | KM677821 |
| Rosario_1316 | FCB | KM677714 | KM677822 |
| Rosario_1317 | FCB | KM677715 | KM677823 |
| Rosario_1318 | FCB | KM677716 | KM677824 |
| Rosario_1320 | FCB | KM677717 | KM677825 |
| Rosario_2137 | FCB | KM677718 | KM677826 |
| Rosario_2138 | FCB | KM677719 | KM677827 |
| BR290_2181 | BR290 | KM677720 | KM677828 |
| BR290_2182 | BR291 | KM677721 | KM677829 |
| BR290_2183 | BR292 | KM677722 | KM677830 |
| BR290_2184 | BR293 | KM677723 | KM677831 |
| BR290_2185 | BR294 | KM677724 | KM677832 |
| BR290_2186 | BR295 | KM677725 | KM677833 |
| BR290_2187 | BR296 | KM677726 | KM677834 |
| BR290_2411 | BR297 | KM677727 | KM677835 |
| BR290_2412 | BR298 | KM677728 | KM677836 |
| BR290_2414 | BR299 | KM677729 | KM677837 |
| CTigre_1810 | CTI | KM677730 | KM677838 |
| CTigre_1811 | CTI | KM677731 | KM677839 |
| CTigre_1812 | CTI | KM677732 | KM677840 |
| CTigre_1815 | CTI | KM677733 | KM677841 |
| CTigre_1816 | CTI | KM677734 | KM677842 |
| CTigre_1817 | CTI | KM677735 | KM677843 |
| CTigre_1818 | CTI | KM677736 | KM677844 |
| CTigre_1819 | CTI | KM677737 | KM677845 |
| CTigre_1820 | CTI | KM677738 | KM677846 |
| CTigre_1288 | CTI | KM677739 | KM677847 |
| FazendaSF_2383 | FSF | KM677740 | KM677848 |
| FazendaSF_635 | FSF | KM677741 | KM677849 |
| CVerdes_999 | CVE | KM677742 | KM677850 |
| CVerdes_986 | CVE | KM677743 | KM677851 |
| CVerdes_987 | CVE | KM677744 | KM677852 |
| CVerdes_638 | CVE | KM677745 | KM677853 |
| CVerdes_2710 | CVE | KM677746 | KM677854 |
| CVerdes_2711 | CVE | KM677747 | KM677855 |
| CVerdes_2713 | CVE | KM677748 | KM677856 |
| CVerdes_2715 | CVE | KM677749 | KM677857 |
| CVerdes_2718 | CVE | KM677750 | KM677858 |
| CVerdes_2722 | CVE | KM677751 | KM677859 |
| Artigas1_2549 | ART1 | KM677752 | KM677860 |
| Artigas1_2550 | ART1 | KM677753 | KM677861 |
| Artigas1_2559 | ART1 | KM677754 | KM677862 |
| Artigas1_2577 | ART1 | KM677755 | KM677863 |
| Artigas1_2578 | ART1 | KM677756 | KM677864 |
| Artigas1_2556 | ART1 | KM677757 | KM677865 |
| Artigas1_2560 | ART1 | KM677758 | KM677866 |
| Artigas1_2564 | ART1 | KM677759 | KM677867 |
| Artigas1_2576 | ART1 | KM677760 | KM677868 |
| Artigas2_2620 | ART2 | KM677761 | KM677869 |
| Artigas2_2621 | ART2 | KM677762 | KM677870 |
| Artigas2_2622 | ART2 | KM677763 | KM677871 |
| Artigas2_2629 | ART2 | KM677764 | KM677872 |
| Artigas2_2630 | ART2 | KM677765 | KM677873 |
| Artigas2_2631 | ART2 | KM677766 | KM677874 |
| Artigas2_2632 | ART2 | KM677767 | KM677875 |
| Artigas2_2633 | ART2 | KM677768 | KM677876 |
| Rivera_2565 | RIV | KM677769 | KM677877 |
| Rivera_2566 | RIV | KM677770 | KM677878 |
| Rivera_2567 | RIV | KM677771 | KM677879 |
| Rivera_2568 | RIV | KM677772 | KM677880 |
| Rivera_2569 | RIV | KM677773 | KM677881 |
| Rivera_2570 | RIV | KM677774 | KM677882 |
| Paysandu_2586 | PAY | KM677775 | KM677883 |
| Paysandu_2589 | PAY | KM677776 | KM677884 |
| Paysandu_2590 | PAY | KM677777 | KM677885 |
| Paysandu_2592 | PAY | KM677778 | KM677886 |
| Paysandu_2593 | PAY | KM677779 | KM677887 |
| Paysandu_2594 | PAY | KM677780 | KM677888 |
| Paysandu_2595 | PAY | KM677781 | KM677889 |
| Paysandu_2596 | PAY | KM677782 | KM677890 |
| Paysandu_2601 | PAY | KM677783 | KM677891 |
| Paysandu_2602 | PAY | KM677784 | KM677892 |
| Salto_2608 | SAL | KM677785 | KM677893 |
| Salto_2610 | SAL | KM677786 | KM677894 |
| Salto_2611 | SAL | KM677787 | KM677895 |
| Salto_2612 | SAL | KM677788 | KM677896 |
| Salto_2613 | SAL | KM677789 | KM677897 |
| Salto_2614 | SAL | KM677790 | KM677898 |
| Salto_2615 | SAL | KM677791 | KM677899 |
| Salto_2616 | SAL | KM677792 | KM677900 |
| Salto_2617 | SAL | KM677793 | KM677901 |
| Salto_2619 | SAL | KM677794 | KM677902 |
| *H. fasciata* | outgroup | KM677795 | KM677903 |
| *H. borellii* | outgroup | KM677796 | KM677904 |
